# Flavonoids Derived from *Opuntia ficus-indica* Fruit Alleviate Renal Injury in Diabetic Nephropathy Mice by Altering Gut Microbiota and Promoting the Production of SCFAs

**DOI:** 10.3390/nu17111800

**Published:** 2025-05-26

**Authors:** Haiping Liao, Yunyi Zhao, Yongheng Liang, Kang Zou

**Affiliations:** 1 College of Life Sciences, Key Laboratory of Agricultural Environmental Microbiology of Ministry of Agriculture and Rural Affairs, Nanjing Agricultural University, Nanjing 210095, China; liaohaiping216@163.com (H.L.); liangyh@njau.edu.cn (Y.L.); 2 College of Animal Science and Technology, Nanjing Agricultural University, Nanjing 210095, China; kangzou@njau.edu.cn; 3 Stem Cell Research and Translation Center, Nanjing Agricultural University, Nanjing 210095, China

**Keywords:** diabetic nephropathy, gut microbiota, short-chain fatty acids

## Abstract

Diabetic nephropathy (DN) represents a severe microvascular complication of diabetes mellitus with limited therapeutic options, many of which are accompanied by considerable adverse effects. *Opuntia ficus-indica* (OFI) fruit, rich in vitamins, dietary fiber, and fatty acids, contains numerous bioactive compounds, including phytosterols, polysaccharides, and flavonoids that demonstrate significant potential in diabetes management. The flavonoid fraction derived from OFI fruit (OFI-F) has exhibited pronounced anti-inflammatory, antioxidant, and gut microbiota modulatory properties. However, the efficacy of OFI-F in ameliorating DN and its underlying mechanisms remain inadequately elucidated. This investigation examined the therapeutic potential of OFI-F in DN and explored its mechanistic pathways. Our findings demonstrate that OFI-F administration significantly attenuated renal injury and intestinal barrier dysfunction in the DN murine model. OFI-F intervention resulted in multiple beneficial outcomes in DN mice, including the mitigation of weight loss, reduction in hyperglycemia, decrease in renal coefficient index, and the attenuation of renal injury. An analysis of gut microbiota composition revealed that OFI-F administration favorably modulated the intestinal microbial community by enhancing the abundance of beneficial bacteria while concomitantly reducing populations of potentially pathogenic bacteria. Additionally, OFI-F treatment promoted the production of short-chain fatty acids (SCFAs), which contributed substantially to renoprotection and inflammatory resolution. Antibiotic intervention studies further confirmed the indispensable role of gut microbiota in mediating the renoprotective effects of OFI-F. In conclusion, this study provides compelling evidence supporting the therapeutic potential of OFI-F in DN management through the concurrent modulation of gut microbiota and renal function, offering a promising nutraceutical approach for alleviating renal injury in DN.

## 1. Introduction

Diabetic nephropathy (DN) is a severe microvascular complication of diabetes and constitutes the predominant cause of end-stage renal disease (ESRD) [1,2]. Clinically, DN is characterized by increased urinary protein excretion, hyperlipidemia, hyperglycemia, and a rapid decline in renal function [3,4]. The pathogenesis of renal injury in DN involves multiple factors, including oxidative stress, lipid accumulation, inflammation, and fibrosis [5,6]. Current therapeutic strategies for early-stage DN primarily focus on glycemic control and blood glucose stabilization. Clinical studies have confirmed the use of sodium–glucose cotransporter 2 (SGLT2) inhibitors as a first-line renoprotective therapy for any chronic kidney disease. SGLT2 inhibitors have cardiovascular and renal protective effects in both diabetic and non-diabetic patients with chronic kidney disease, in addition to improving glycemic control in patients with DN [7]. However, these pharmacological interventions are associated with high costs, and the long-term use of hypoglycemic drugs will lead to adverse side effects, including drug resistance, adverse reactions, and even toxicity [8,9]. Consequently, an improved understanding of chronic kidney disease pathogenesis is needed to optimize treatment. The use of natural products effective in diabetes treatment may reduce drug intake through dietary interventions. The consumption of vegetables and fruits rich in anthocyanins, dihydroflavonols, catechins, and flavonols has been reported to inhibit glucosidase and reduce the risk of diabetes. These findings underscore the potential of natural products as promising agents for diabetes prevention and management [10].

Disruption of the ecological homeostasis of the gut microbiota is strongly associated with the development of diabetes. An imbalance in the ecological balance of the gut microbiota affects insulin resistance, the metabolism of SCFAs, and the gut barrier [11,12]. Studies on the gut–renal axis have shown that intestinal function affects not only the composition of the gut microbiota but also the renal metabolic and immune microenvironment. This interaction through the gut–renal axis may play an important role in the onset and development of DN [13]. Therefore, the treatment of DN from the perspective of gut microbiota is also a promising approach.

OFI is a dicotyledonous angiosperm plant native to tropical and subtropical regions. It has been shown that OFI fruit has various pharmacological properties, including anti-inflammatory, antioxidant, anti-bacterial, anti-diabetic, and anti-tumor [14]. Recent studies have demonstrated the effectiveness of OFI juice as a complementary diabetes treatment and have recommended that the flavonoids in OFI juice be included in standard treatment [15]. OFI fruit is the reproductive organs of the plant and is rich in nutritive active ingredients, notably in antioxidants like polyphenols and flavonoids [16]. However, research on the efficacy of OFI-F is still in its infancy. The evaluation of the efficacy and potential mechanisms of flavonoid components in diabetic mice still needs to be further explored and investigated.

In this study, a flavonoid mixture at 90% purity was extracted from OFI fruit to investigate its effects on the DN model in mice induced by streptozotocin (STZ) and a high-fat diet (HFD). The findings from this study establish a scientific foundation for the potential clinical application of OFI-derived flavonoids in DN management.

## 2. Materials and Methods

### 2.1. Preparation of OFI-F

The basic processes by which the company (DASF Biotechnology Co., Ltd., Nanjing, China) carries out preparation of OFI-F: the OFI fruit raw material was crushed using a universal grinding machine (Yong guang ming Medical Equipment Factory, Beijing, China), followed by the addition of 60% ethanol and stirring for extraction at 40 °C. At the end of the process, filtration and concentration were carried out, and, then, water was added for overnight standing. The next day, centrifugation was performed, and the supernatant was discarded. The remaining precipitate was extracted with a mixture of ethyl acetate and petroleum ether with stirring. After 12 h, the precipitate recovered by concentration after filtration was subjected to petroleum ether to remove impurities and then filtered and dried again. The precipitate was then dissolved in ethanol and 2 times added to silica gel for stirring and drying. Finally, the mixture was eluted using ethyl acetate and petroleum ether, and the eluate was collected. The collected solution was centrifuged at 2000× *g* for 15 min to obtain a precipitate. The precipitate was washed with distilled water and left to stand overnight, yielding the final product, OFI-F. According to the data provided by DASF Biotechnology, the material used in the experiment was prepared from 5 kg of dry weight OFI, 12.3 g of OFI-F was obtained in the end, and the yield of OFI-F was 0.246%. A flow chart illustrating the separation and purification process of OFI is shown in Supplementary Figure S1.

### 2.2. Animal Models

C67BL/6J (8-week-old male) was purchased from the Experimental Animal Center of Nanjing Agricultural University. Mice were housed under environmental conditions (temperature: 25 °C ± 0.5 °C, humidity: 55% ± 5%, and 12 h light/dark cycle), with free access to food and water throughout the experiments. The experimental procedures were approved by the Experimental Animal Center of Nanjing Agricultural University (Approval No (NJAU.No20240817151)), and all experimental operations were carried out following the guidelines established by the committee. Mice were fed either a standard normal control diet (XTI01WC-009, JSXTSW, Yangzhou, China) or an HFD (XTM01-002, JSXTSW, Yangzhou, China) throughout this study.

### 2.3. Drugs and Chemicals

The following drugs and chemicals were used: OFI-F (DASF Biotechnology Co., Ltd., Nanjing, China), STZ (Macklin, Shanghai, China), 0.1 M sodium citrate buffer (pH4.5, Beyotime, Shanghai, China), 95% stroke-physiological saline solution (Beyotime, Shanghai, China), D-( + )-glucose (Beyotime, Shanghai, China), Dulbecco’s modified Eagle’s medium (DMEM) (Hyclone, Logan, UT, USA), 10% fetal bovine serum (ExCell Bio, Shanghai, China), 100 ug/mL of penicillin and 100 μg/mL of streptomycin (Beyotime, Shanghai, China), lipopolysaccharide (LPS) (Beyotime, Shanghai, China), TRIZOL reagent (TSINGKE, Nanjing, China), RIPA lysis buffer (Biosharp, Anhui, China), β-Tubulin Rabbit mAb (A12289, ABclonal, Wuhan, China), nuclear factor κB (NF-κB) p65/RelA Rabbit mAb (A19653, ABclonal, Wuhan, China), Phospho-NF-κB p65/RelA-S536 Rabbit mAb (AP1294, ABclonal, Wuhan, China), Toll-like receptor 4 (TLR4) (A11226, ABclonal, Wuhan, China), myeloid differentiation primary response 88 (MYD88) (A21905, ABclonal, Wuhan, China), CD68 (A20803, ABclonal, Wuhan, China), Zonula occluden-1 (ZO-1) (A0659, ABclonal, Wuhan, China), Occludin (A12621, ABclonal, Wuhan, China).

### 2.4. Experimental Design

After 1 week of acclimatization, forty-two mice were randomly divided into the following seven groups (*n* = 6). During the non-antibiotic treatment, the control group was provided with filtered tap water, the model group and the treatment group were given high-glucose drinking water and HFD, and STZ was injected intraperitoneally (50 mg/kg) for five consecutive days. The selection of drug treatments for mice and the selection of treatment concentrations were conducted according to previous reports [17]. Both the control and model groups were administered normal saline daily via oral gavage, whereas the treatment group was further subdivided into low-dose and high-dose groups. The low-dose group was daily gavaged with 300 mg/kg of OFI-F, and the high-dose group was daily gavaged with 500 mg/kg of OFI-F. The mice were sacrificed after 3 weeks, and the feces, blood, and tissue samples were collected. The mice were anesthetized with pentobarbital and euthanized by cardiac puncture under anesthesia. All procedures were performed in accordance with relevant ethical guidelines. During the antibiotic interference treatment, after 2 weeks of the HFD, the drinking water was changed to an antibiotic-mixed aqueous solution (ABX: ampicillin (1 g/L); neomycin sulfate (1 g/L); metronidazole (1 g/L); and vancomycin (0.5 g/L)) for gut microbiota removal. In the antibiotic-treated group, the treatment was the same as in the non-antibiotic group, except that the drinking water was different.

### 2.5. Induction of DN in Mice Model

According to previous studies, DN was induced by using STZ combined with an HFD. For a 2-week HFD period, after 12 h fasting, HFD mice were intraperitoneally injected with STZ (STZ was dissolved in a 0.1 mol/L sodium citrate buffer, and the concentration was adjusted to 10 mg/mL and sterilized using a 0.22 μm filter. The actual injection volume was calculated at a dose of 50 mg/kg per mouse, intraperitoneal injection, 5 days) to induce the DN model at the end of week 2 (day 14). Then, 72 h post-injection, fast blood glucose levels were measured by a Yuwell 520 glucometer (Shanghai, China). Levels of blood glucose > 13.8 mmol/L were considered a sign of diabetes, and the animals were used for the next treatment and investigations.

### 2.6. Genomics DNA Graxtraction and 16S rRNA Gene Pyrosequencing

The microbial community DNA was extracted using MagPure Stool DNA KF kit B (Magen, Guangzhou, China) following the manufacturer’s instructions. DNA was quantified with a Qubit Fluorometer by using a Qubit dsDNA BR Assay kit (Invitrogen, Carlsbad, CA, USA), and the quality was checked by running an aliquot on 1.8% agarose gel (Beijing Bomei Fuxin Technology Co., Ltd., Beijing, China). Variable regions V3–V4 of the bacterial 16S rRNA gene were amplified with degenerate PCR primers, 338F (5’ACTCCTACGGGAGGCAGCA-3’) and 806R (5’- GGACTACHVGGGTWTCTAAT-3’). Both forward and reverse primers were tagged with Illumina adapters, pads, and linker sequences. PCR enrichment was performed in a 50 μL reaction containing a 30 ng template, fusion PCR primer, and PCR master mix. PCR cycling conditions were as follows: 94 °C for 3 min, 30 cycles of 94 °C for 30 s, 56 °C for 45 s, 72 °C for 45 s, and final extension for 10 min at 72 °C for 10 min. The PCR products were purified with AmpureXP beads and eluted in Elution buffer. For sequencing of the constructed library, the Illumina NovaSeq 6000 sequencing platform was used, employing a paired-end (PE) 250 sequencing strategy. Raw reads were filtered to remove adaptors and low-quality and ambiguous bases, and then paired-end reads were added to tags by the Fast Length Adjustment of Short reads program (FLASH, v1.2.11) to obtain the tags. OTU clustering was chosen for third-generation sequencing data. OTU Clustering: VSEARCH (version 2.4.3) performs sequence clustering at a default similarity level of 97%. Low Abundance Filtering: After obtaining the initial OTU/ASV abundance table, features with low abundance are filtered out. The filtering criteria are automatically adjusted based on the sample characteristics. Alpha and beta diversity were estimated by QIIME2 (https://qiime2.org, accessed on accessed on 26 October 2024) at the OTU level, respectively. Barplot and heatmap of different classification levels were plotted with the R package v3.4.1 and the R package “gplots”, respectively. Principal component analysis (PCA) in OTUs was plotted with R package “ade4”. Species accumulation curves were plotted with R package version 3.1.1, developed by BGI tech (Wuhan, China).

### 2.7. SCFAs Measurement

Samples were homogenized for 1 min with 500 μL of water and 100 mg of glass beads, and then they were centrifuged at 4 °C for 10 min at 14,000× *g*. A 200 μL supernatant was extracted with 100 μL of 15% phosphoric acid and 20 μL of 375 μg/mL 4-methylvaleric acid solution and 280 μL of diethyl ether. Subsequently, the samples were centrifuged at 4 °C for 10 min at 14,000× *g* after vortexing for 1 min, and the supernatant was transferred into the vial prior to gas chromatography–mass spectrometry (GC–MS) (GC2030-QP2020, Shimadzu, Co., Ltd., Kyoto, Japan) analysis.

### 2.8. Cell Culture

Human kidney epithelial cells (Hkb-20) (YS1195C, Yage Biotechnology Co., Shanghai, China), cell lines derived from proximal tubule epithelium that play a key role in renal reabsorption and secretion processes [18], were cultured in DMEM, supplemented with 10% fetal bovine serum, 100 μg/mL of penicillin, and 100 μg/mL of streptomycin. The cells were incubated at 37 °C with 5% CO_2_. Hkb-20 cells were treated with 10 ng/mL of LPS for 6 h and then treated with low, medium, and high concentration gradients of OFI-F (100 mg/mL, 300 mg/mL, 500 mg/mL). After 18 h, the cells were collected.

### 2.9. CCK8 (Cell Counting Kit-8) Assay

Cell viability was analyzed by Cell Counting Kit-8 (YiSheng, Shanghai, China) according to the manufacturer’s protocols. The transfected cells were plated into 96-well plates (Corning Costar, Corning, NY, USA) at a density of 5.0 × 10^3^/well/100 µL. Then, the cells were treated with various concentrations of OFI-F (0, 100, 300, and 500 mg/mL). After treatment for 48 h, 10 µL of CCK8 solution was added to each well, followed by incubation for 2 h. The absorbance was analyzed at 450 nm using a microplate reader (Bio-Rad, Hercules, CA, USA) using wells without cells as blanks. The proliferation of cells was expressed by the absorbance. Cell inhibition was calculated from the absorbance obtained. Inhibition rate (%) = [(Ac − As)/(Ac − Ab)] × 100%. As is the absorbance of experimental wells, Ac is the absorbance of control wells, and Ab is the absorbance of blank wells.

### 2.10. Fasting Blood Sugar and Glucose Tolerance Test (GTT)

Fasting blood glucose levels were measured using the Yuwell 520 glucometers (Shanghai, China) from tail vein blood samples every 3 days. If the fasting blood sugar level (fasting time does not exceed 6 h) is >11.1 mmol/L, and the random blood sugar is >16.7 mmol/L, it is considered to have reached the disease standard. Mice were fasted overnight for 16 h at age 8 weeks for GTT. After intraperitoneal injection of 10% glucose solution (15 mg/kg), GTT was performed with a collection of tail blood glucose at 0, 15, 30, 90, and 120 min after injection, respectively.

### 2.11. Measurement of Bcr, BUN, TC, and TG

Blood creatinine (Bcr) and blood urea nitrogen (BUN) levels are the most common indicators of renal function. Blood samples were collected from the orbital sinus of mice, and serum was extracted after centrifugation (1000× *g* for 15 min at 4 °C). Bcr and BUN were quantified according to the manufacturer’s instructions (Detect X Serum Creatinine Detection Kit, Arbor Assays, Ann Arbor, MI, USA; QuantiChrom Urea Assay Kit, BioAssay Systems, Hayward, CA, USA, respectively). For Bcr quantification, the Arbor Assays Detect X Serum Creatinine Detection Kit employs an enzymatic method that avoids interference from non-creatinine chromogens, offering improved specificity over traditional Jaffe methods, per the manufacturer’s protocol and Osmic-Husni et al. (2023) [19]. These kits are selected for their reproducibility in murine models, as corroborated by studies such as Davis et al. (2020) in J Vet Diagn Invest [20]. Triglyceride (TG) and total cholesterol (TC) are key indicators of lipid metabolism. According to the manufacturer’s instructions, TG (A110-1-1, Nanjing Jiancheng Bioengineering Institute, Nanjing, China) and TC (A111-1-1, Nanjing Jiancheng Bioengineering Institute, Nanjing, China) kits were used to determine the levels of TG and TC, respectively [21].

### 2.12. Measurement of MDA, CAT, SOD, and GSH

Cells were incubated for 18 h under the following conditions: LPS (10 ng/mL), LPS + OFI-F-L (100 mg/mL), LPS + OFI-F-M (300 mg/mL), and LPS + OFI-F-H (500 mg/mL). Following incubation, cellular levels of malondialdehyde (MDA), catalase (CAT), superoxide dismutase (SOD), and glutathione (GSH) were quantified using commercial assay kits (Nanjing Jiancheng Bioengineering Institute, Nanjing, China). These measurements were performed according to established protocols [22,23]. A 10% kidney homogenate was used to measure the activities of MDA, CAT, and SOD and the content of GSH. The activities of MDA (A003-1-2), CAT (A007-1-1), SOD (A001-3-2), and the GSH content (A005-1-2) were determined spectrophotometrically using commercial kits (Nanjing Jiancheng Bioengineering Institute, Nanjing, China) according to the procedures of Kok Poh Loh et al. [24], Jia-Qing Zhang et al. [25], Shao-Hua Liu et al. [26], and Yan Deng et al. [27], respectively. All measurements were conducted spectrophotometrically using the same commercial kits for both cellular and kidney homogenate samples, following the methodologies described in the respective references.

### 2.13. Measurement of IL-6 and TNFα

Cells were incubated for 18 h under the following conditions: LPS (10 ng/mL), LPS + OFI-F-L (100 mg/mL), LPS + OFI-F-M (300 mg/mL), and LPS + OFI-F-H (500 mg/mL). Following incubation, cellular levels of interleukin 6 (IL-6) and tumor necrosis factor alpha (TNFα) were quantified using commercial assay kits (Nanjing Jiancheng Bioengineering Institute, Nanjing, China). These measurements were performed according to established protocols [28]. A 10% kidney homogenate was used to measure the activities of IL-6 and TNFα. The activities of IL-6 (H007-1-2) and the TNFα content (H052-1-2) were determined spectrophotometrically using commercial kits (Nanjing Jiancheng Bioengineering Institute, Nanjing, China) according to the procedures of Zhang et al. [29].

### 2.14. Quantitative Real-Time PCR

Total RNA was extracted from tissues (colon and kidney) or cultured cells using TRIZOL reagent (TSINGKE, Nanjing, China) according to the manufacturer’s instructions, and the concentration and purity of total RNA were detected. Then, the total RNA was reverse transcribed into cDNA using the Servicebio RT First Strand cDNA Synthesis Kit (Servicebio, Wuhan, China) and stored at −80 °C. qPCR was performed on the QuantStudio 6 Flex Real-Time PCR System, and all steps were performed according to the 2X SYBR Green QPCR Master Mix kit (Servicebio, Wuhan, China) instructions. The following thermal program was applied: firstly, predenaturation at 95 °C for 30 s, followed by 40 amplification cycles of 15 s denaturing step (95 °C), 10 s annealing–extension step (60 °C), 30 s extending step (72 °C) to obtain SYBR^®^ Green fluorescence signal. Relative expression of mRNA was analyzed using the 2^−ΔΔct^ method, and Ct values were normalized to glyceraldehyde 3-phosphate dehydrogenase (GAPDH). Primer sequences for RT-qPCR analysis are shown in Table 1 and Table 2.

### 2.15. Western Blotting Analysis

Submerging the tissues (colon and kidney) in liquid nitrogen will freeze the tissue pieces most quickly. The frozen samples were powdered by grinding the frozen tissue fragments in a prechilled mortar. Total protein was isolated from the samples using RAPI lysis buffer (Beyotime, Biotech, Shanghai, Jiangsu, China). Cell samples were directly collected with an appropriate amount of lysis buffer according to cell density. After quantifying the total protein concentration using a bicinchoninic acid (BCA) assay, the samples were separated by 10% SDS-PAGE and subsequently transferred to a polyvinylidene fluoride (PVDF) membrane. Then, the membrane was blocked in phosphate-buffered saline tween 20 (PBST) with 5% skim milk for 1 h, incubated with primary antibodies overnight at °4 C. Next, horseradish peroxidase (HRP)-conjugated secondary antibodies were incubated for 1 h. Chemical imaging was performed with an ultrasensitive ECL chemiluminescence reagent. Band intensities were quantified using ImageJ 1.50i software (National Institutes of Health, Bethesda, MD, USA).

### 2.16. Histology Assessment and Immunohistochemical Analysis

The renal and colon tissues were harvested and fixed in 4% phosphate-buffered paraformaldehyde for 48 h at 4 °C. After embedding in paraffin, sections (5 μm) were prepared, deparaffinized in xylene, and rehydrated in a series of graded ethanol solutions. For general histology, sections were processed for staining according to the manufacturer’s instructions (Solarbio Life Science, Beijing, China). All tissues were subject to Hematoxylin and Eosin staining (HE). Renal sections were also performed for periodic acid–Schiff stain (PAS) for polysaccharides and mucosubstances, Masson stain for collagen and muscle fibers, and Picro-Sirius Red Stain (SR) for collagen. For immunohistochemistry staining, sections were incubated with primary antibody against CD68 (sc-20060, 1:100, Santa Cruz Biotechnology, Dallas, TX, USA) and then followed by incubation with secondary antibodies and the use of diaminobenzidine substrate kit (Servicebio, Shanghai, China).

### 2.17. FITC Dextran Gut Permeability

The gut tissue sample was diluted 1:100 (*v*/*v*) in PBS for analysis using 4-kDa fluorescein isothiocyanate (FITC)-dextran. FITC fluorescence was measured in duplicate using a black 96-well plate (Corning, Corning, NY, USA) and a microplate spectrophotometer (Bio-Rad, Hercules, CA, USA). The excitation wavelength was set at 485 nm, and the emission was measured at 535 nm. A standard curve was generated using FITC-dextran concentrations ranging from 0 to 25 µg/mL in PBS, which allowed for the determination of sample concentrations based on this curve.

### 2.18. Statistical Assessment

The objective of this investigation was to examine the differences in structural divergence in the 16S rRNA gene sequence data, in addition to evaluating the alpha diversity indexes and weighted UniFrac distances. The significance of these variances was assessed using a Kruskal–Wallis rank–sum test. All analyses were conducted in R version 4.2.0 (BGI Tech, Wuhan, China). Data are presented as mean ± standard error of the mean (SEM). Statistical significance (* *p* < 0.05, ** *p* < 0.01, *** *p* < 0.001, **** *p* < 0.0001; n.s., not significant) by one-way ANOVA followed by Tukey’s multiple comparison test or Kruskal–Wallis test followed by Dunn’s multiple comparison test (for qPCR results) in GraphPad Prism 9 (GraphPad Software, San Diego, CA, USA).

## 3. Results

### 3.1. OFI-F Alleviated LPS-Induced Inflammation

**Figure 1:**
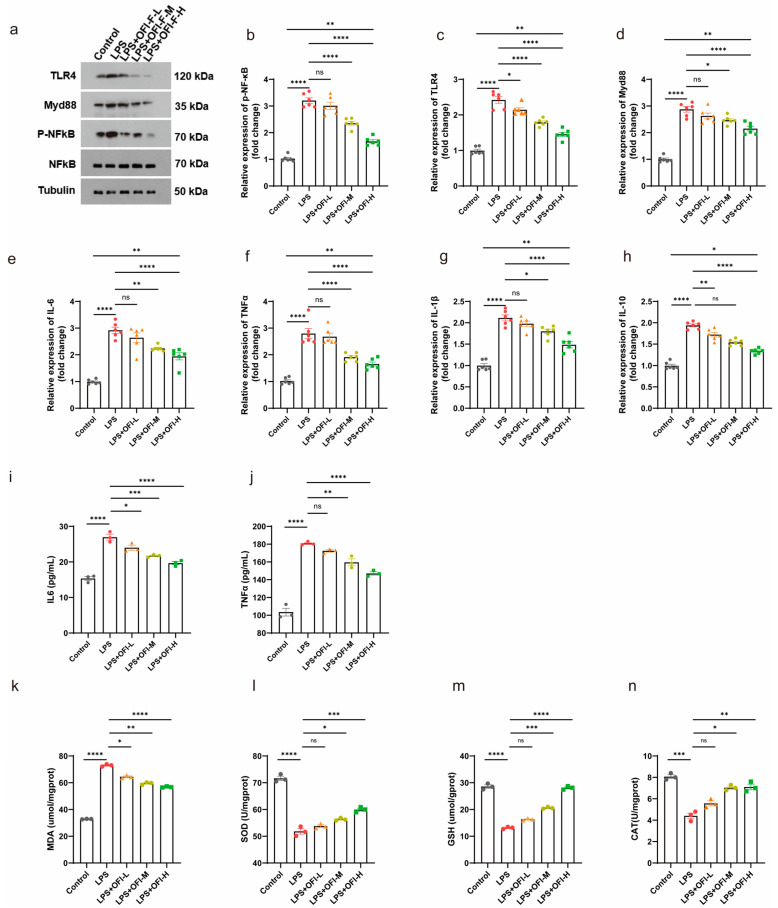
OFI-F inhibits LPS-induced inflammation in renal cells: (**a**) Western blot analysis of TLR4/NF-κB signaling pathway. *NF-κB* (**b**), *TLR4* (**c**), *MYD88* (**d**), *IL-6* (**e**), *TNFα* (**f**), *IL-1β* (**g**), and *IL-10* (**h**) mRNA expressions were detected by fluorescence quantitative PCR (*n* = 6). GAPDH is selected as the internal standard. Determination of IL-6 (**i**), TNFα (**j**), MDA (**k**), SOD (**l**), GSH (**m**), and CAT (**n**) concentrations in Hkb-20 cells by ELISA. LPS-induced Hkb-20 cells are treated with varying concentrations of OFI-F: 0 mg/mL (control, PBS), 100 mg/mL (L), 300 mg/mL (M), and 500 mg/mL (H). This experimental setup is designed to create inflammatory environments and to evaluate the effects of OFI-F treatment on protein and mRNA levels in these conditions. Hkb-20 cells without LPS induction serve as a control. Data are presented as mean ± SEM (*n* = 3). Statistical significance (* *p* < 0.05, ** *p* < 0.01, *** *p* < 0.001, **** *p* < 0.0001; ns, not significant) is determined by one-way ANOVA followed by Tukey’s multiple comparison test or Kruskal–Wallis test followed by Dunn’s multiple comparison test (for qPCR results).

In vitro studies have found that flavonoids in OFI fruit have anti-inflammatory and antioxidant physiological activities [16]. To evaluate the bioactivity of OFI-F, we utilized an LPS-induced inflammatory renal cell model, analyzing its effects on key proteins in the TLR4/NF-κB signaling pathway and the regulation of relevant inflammatory cytokines. As shown in Figure 1a, treatment with LPS resulted in an increase in the phosphorylation of NF-κB, as well as elevated levels of TLR4 and MYD88 expression. However, the administration of OFI-F at concentrations of 100 mg/mL (L), 300 mg/mL (M), and 500 mg/mL (H) significantly reduced these expression levels in a dose-dependent manner. Additionally, the results of the mRNA relative expression analysis corresponded with the findings from the immunoblotting (Figure 1b–d). It is important to note that CCK-8 assays revealed that OFI-F did not exhibit a significant inhibitory effect on cell proliferation within our selected concentration range (Figure S2). Further experiments revealed that OFI-F treatment dose-dependently inhibited the mRNA expression of key pro-inflammatory cytokines (*IL-6*, *TNFα*, *Interleukin-1 beta* (*IL-1β*) and *Interleukin 10* (*IL-10*)) in LPS-stimulated cells (Figure 1e–h) while also decreasing the secretion of IL-6 and TNFα (Figure 1i,j). In addition, OFI-F administration significantly decreased MDA activity and increased the activities of antioxidant enzymes including SOD, CAT, and GSH (Figure 1k–n). Collectively, these results indicate that OFI-F possesses potent anti-inflammatory and antioxidant properties.

### 3.2. OFI-F Improved the Severity of DN in Mice

**Figure 2:**
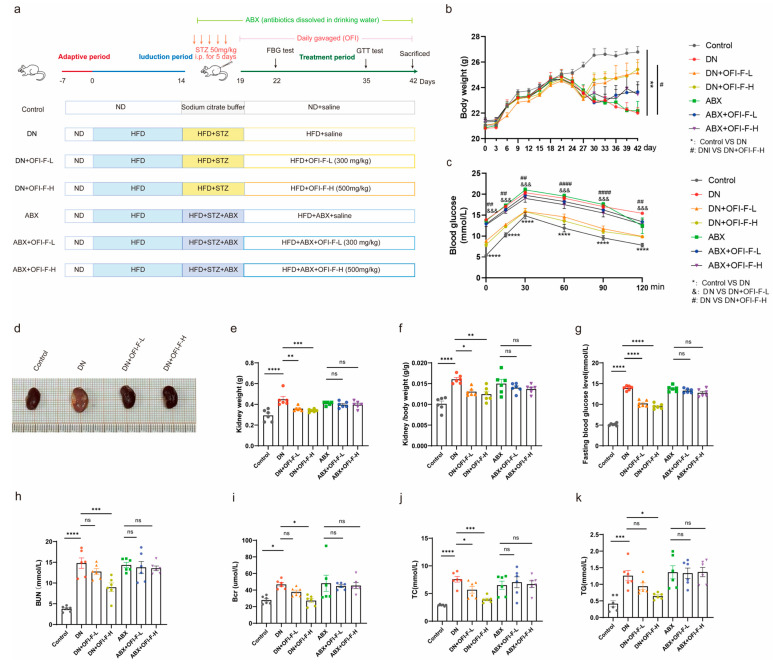
OFI-F treatment attenuates renal injury in DN mice: (**a**) Schematic diagram of the design of an animal experiment. (**b**) Graph of body weight changes in mice during the experiment. (**c**) Glucose tolerance test. (**d**) Representative images of kidney in Control, DN, DN + OFI-F-L, and DN + OFI-F-H group mice at sacrifice. (**e**) Mouse kidney weight in different condition groups. (**f**) Kidney weight-to-body weight ratio in each group. (**g**) Fasting blood glucose in mice. Serum concentrations of Bcr (**h**), BUN (**i**), TC (**j**), and TG (**k**) in mice are measured by ELISA. The treatment concentrations of OFI-F in DN mice are as follows: low dose (L, 300 mg/kg) and high dose (H, 500 mg/kg). Data are presented as mean ± SEM (*n* = 6). Statistical significance (* *p* < 0.05, ** *p* < 0.01, *** *p* < 0.001, **** *p* < 0.0001; ns, not significant) are determined by one-way ANOVA followed by Tukey’s multiple comparison test. &&&, #, ##, #### are used in the same way as the asterisk (*) in the figure (where * *p* < 0.05, ** *p* < 0.01, *** *p* < 0.001, **** *p* < 0.0001). The symbols &&& and *** serve the same purpose but use different markers to distinguish between different comparison groups.

To assess whether OFI-F ameliorates DN by reducing renal injury, we employed an STZ/HFD-induced DN murine model. The establishment of the DN mice model and the administration of OFI-F are shown in Figure 2a. During the experiment, the DN model mice showed significant weight loss and excessive drinking and urination at day 5 after STZ injection. However, these symptoms improved after treatment with OFI-F, administered via gavage at doses of 300 and 500 mg/kg/d (see Figure S3a,b). Consistent with the expected results, DN mice had abnormally higher glucose levels and fasting blood glucose levels than the control group and consistently maintained high blood glucose levels, but the blood glucose clearance rate of DN mice was significantly improved following treatment with OFI-F (Figure 2b–d). However, the hypoglycemic activity of OFI-F disappeared among the ABX-treated groups. In this experiment, we initially observed the general appearance of the renal in DN mice, which showed paleness, edema, and hypertrophy. In contrast, the OFI-F-treated group recovered from redness, swelling, and smoothness (Figure 2e). The renal coefficient, as well as the levels of Bcr and BUN in the DN mice, were significantly higher than those of the control group (Figure 2h,i). In addition, the levels of serum TC and serum TG, which are typical indicators of lipid metabolism, were markedly elevated in the DN mice model. In the OFI-F-treated mice, these indicators were significantly reduced alongside the increased dosage of OFI-F (see Figure 2j,k). These results suggest that OFI-F is effective in mitigating renal injury in DN mice.

### 3.3. OFI-F Effectively Alleviated Renal Injuries in DN Mice

**Figure 3:**
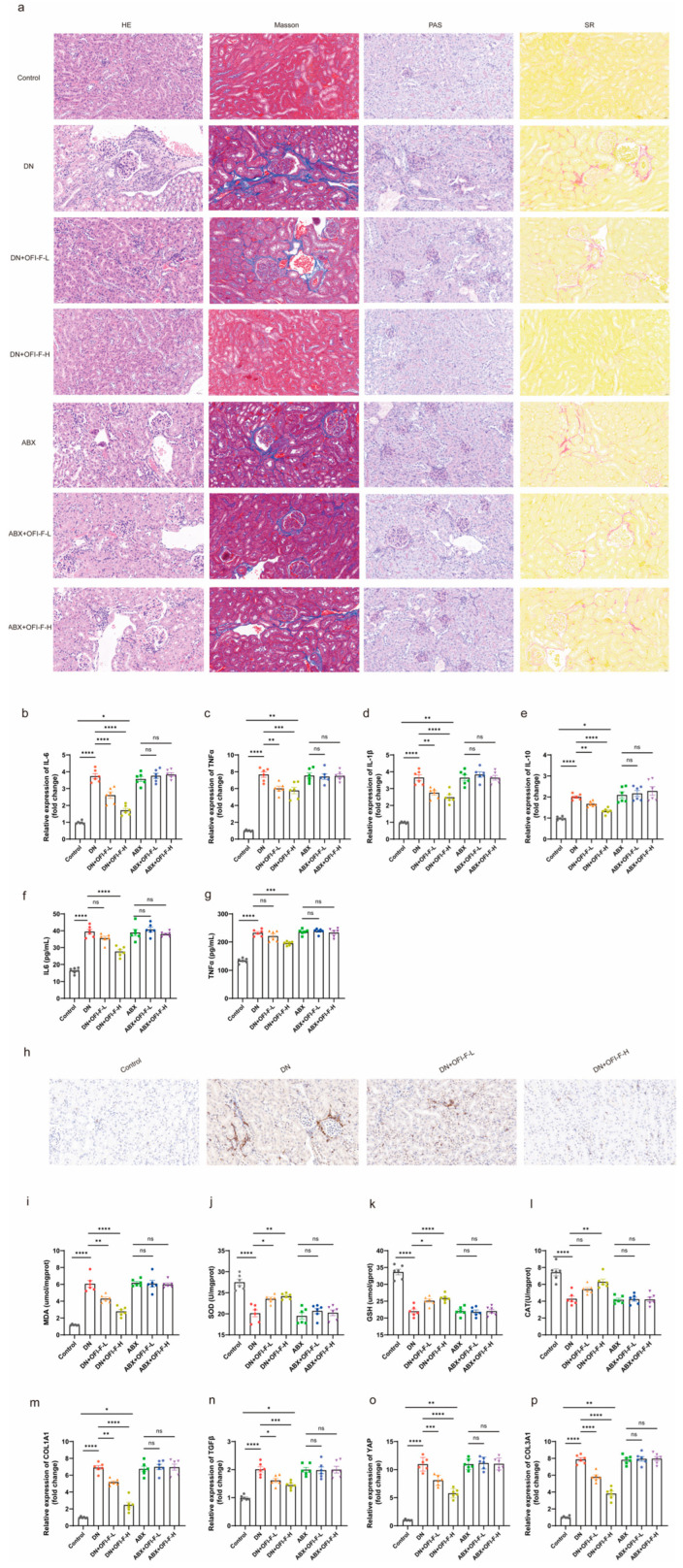
OFI-F improves inflammation, oxidative stress, and fibrosis in DN mice: (**a**) Kidney tissue in HE, Masson, PAS, and SR staining. *IL-6* (**b**), *TNFα* (**c**), *IL-1β* (**d**), and *IL-10* (**e**) are detected by fluorescence quantitative PCR. Determination of IL-6 (**f**) and TNFα (**g**) concentrations in mouse kidney tissues by ELISA. (**h**) Immunohistochemistry of CD68 in mouse kidney tissue. Determination of MDA (**i**), SOD (**j**), GSH (**k**), and CAT (**l**) concentrations in mouse kidney tissues by ELISA. *COL1A1* (**m**), *TGF-β* (**n**), *YAP* (**o**), and *COL3A1* (**p**) mRNA expressions are detected by fluorescence quantitative PCR. GAPDH is selected as an internal standard. The treatment concentrations of OFI-F in DN mice are as follows: low dose (L, 300 mg/kg) and high dose (H, 500 mg/kg). Data are presented as mean ± SEM (*n* = 6). Statistical significance (* *p* < 0.05, ** *p* < 0.01, *** *p* < 0.001, **** *p* < 0.0001; ns, not significant) is determined by one-way ANOVA followed by Tukey’s multiple comparison test or Kruskal–Wallis test followed by Dunn’s multiple comparison test (for qPCR results).

To further explore how OFI-F improves renal injury and exerts kidney protection, we compared the pathological damage in kidney tissues among the different groups of mice. The results indicated that DN mice exhibited glomerular hypertrophy, thickening of the glomerular basement membrane, glycogen accumulation, and fibrosis. However, treatment with OFI-F significantly improved these pathological conditions (Figure 3a). Further studies found that OFI-F treatment alleviated inflammation in DN mice. Specifically, after OFI-F intervention, the mRNA expression levels of *IL-1β*, *IL-6*, *TNFα*, and *IL-10* decreased, the secretion of IL-6 and TNFα in serum decreased, and the number of CD68-positive macrophages in the kidney decreased (Figure 3b–h). Moreover, OFI-F administration significantly ameliorated oxidative stress, as evidenced by a reduction in MDA levels and a marked increase in the activity of antioxidant enzymes, including SOD, CAT, and GSH (Figure 3i–l). Since inflammation and oxidative stress are the main causes of fibrosis [30], combined with Figure 3a and the mRNA expression detection of fibrosis-related factors (C*ollagen Type I Alpha 1 Chain (COL1A1)*, *transforming growth factor beta (TGF-β)*, *yes-associated protein (YAP)*, and *Collagen Type III Alpha 1 Chain (COL3A1)*) (Figure 3m–p), it shows that OFI-F plays a certain role in mitigating fibrosis development in DN mice by attenuating inflammatory responses and enhancing antioxidant defense mechanisms, which are associated with its improvement of renal injury.

### 3.4. OFI-F Improved Gut Microbiota Dysbiosis in STZ/HFD-Induced DN Mice

**Figure 4:**
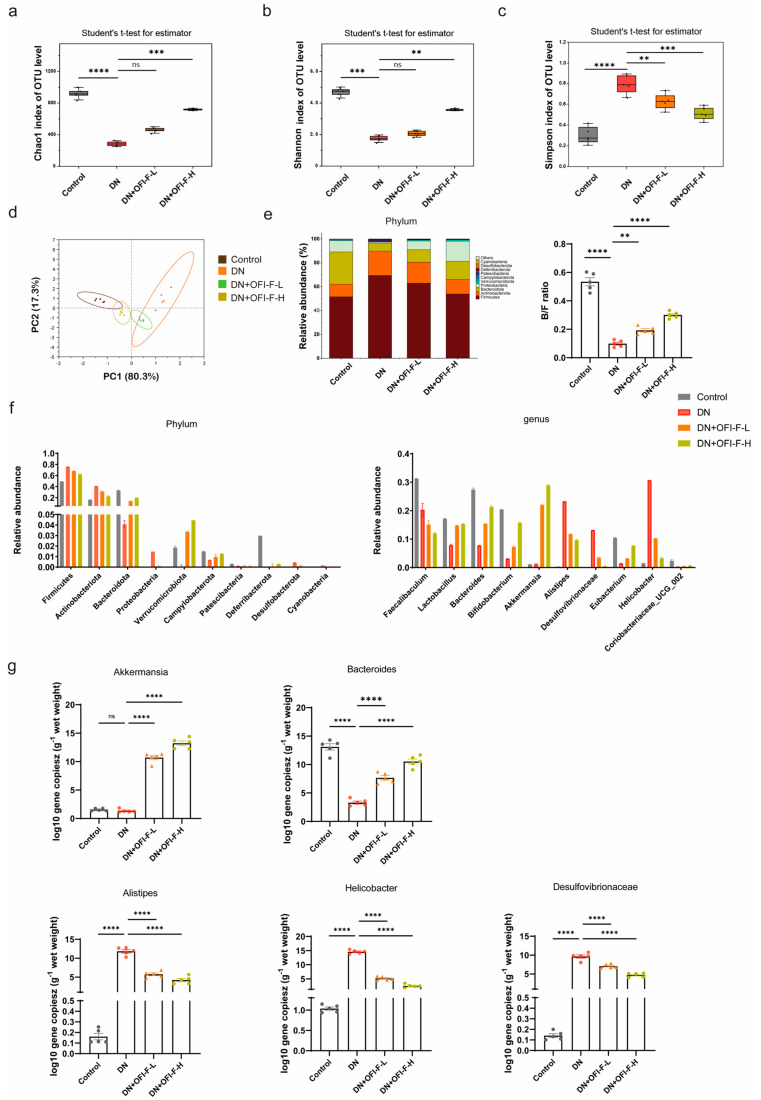
Effects of OFI-F treatment on gut microbiota in DN mice: (**a**) Chao 1 index shows that OFI-F treatment reverses reduced bacterial abundance in DN mice. (**b**) α-Diversity at the operational taxonomic unit (OTU) level estimated by the Shannon index. (**c**) Simpson index shows that OFI-F treatment promotes increased abundance of intestinal bacteria. (**d**) PCA score plots based on unweighted UniFrac analysis of variance are used to test the difference between groups at the OTU level. (**e**) Species accumulation analysis, the ratio of *Bacteroidetes* to *Firmicutes*. (**f**) The relative abundance of bacterial community at the phylum (left) and genus (right) level. (**g**) Absolute quantitative detection of high-abundance bacteria that is significantly altered at the phylum and genus level by OFI-F treatment. Data are presented as mean ± SEM (*n* = 5). The significance of these variances is assessed using a Kruskal–Wallis rank–sum test. Statistical significance (** *p* < 0.01, *** *p* < 0.001, **** *p* < 0.0001; ns, not significant) is determined by one-way ANOVA followed by Tukey’s multiple comparison test.

Previous research results indicated that the renal-protective, anti-inflammatory, and antioxidant activities of OFI-F are associated with gut microbiota. To further assess the influence of OFI-F on gut microbiota composition in DN mice, 16S rRNA sequencing was performed. The results demonstrated that OFI-F treatment enhanced microbial diversity and richness, as evidenced by increased Chao1 and Shannon indices and a decreased Simpson index (Figure 4a–c). Additionally, PCA results showed that there was a significant difference between the DN + OFI-F-H group and the DN group (Figure 4d). Next, we analyzed the gut microbiota at the phylum level. The results showed that the *Actinobacteriota* abundance of the DN + OFI-F -L (19.55%) and DN + OFI-F -H (13.60%) groups were significantly lower than the DN (23.90%) group. The *Bacteroidetes*/*Firmicutes* ratio (B/F ratio) of the OFI-F treatment groups was significantly higher than the DN group (Figure 4e). In addition, the abundance of DN group pathogenic bacteria *Actinobacteriota* increased, while the abundance of *Verrucomicrobiota* also rose at the phylum level (Figure 4f). At the genus level, compared with the DN group, after OFI-F treatment, beneficial bacteria (*Akkermansia* and *Bacteroides*) have increased, while harmful bacteria (*Alistipes, Helicobacter,* and *Desulfovibrio*) have decreased (Figure 4f). Consistently, the absolute quantitative results further showed that the abundance of pathogenic bacteria *Alistipes*, *Helicobacter,* and *Desulfovibrio* was significantly reduced after OFI-F treatment (Figure 4g, *p* < 0.01). The abundance of beneficial bacteria *Akkermansia* and *Bacteroides* increased significantly (Figure 4g, *p* < 0.01). In summary, OFI-F affects the composition of gut microbiota and promotes the increase in beneficial bacteria in the intestine of DN mice.

### 3.5. OFI-F Treatment Modulated Fecal SCFA Levels in DN Mice

**Figure 5:**
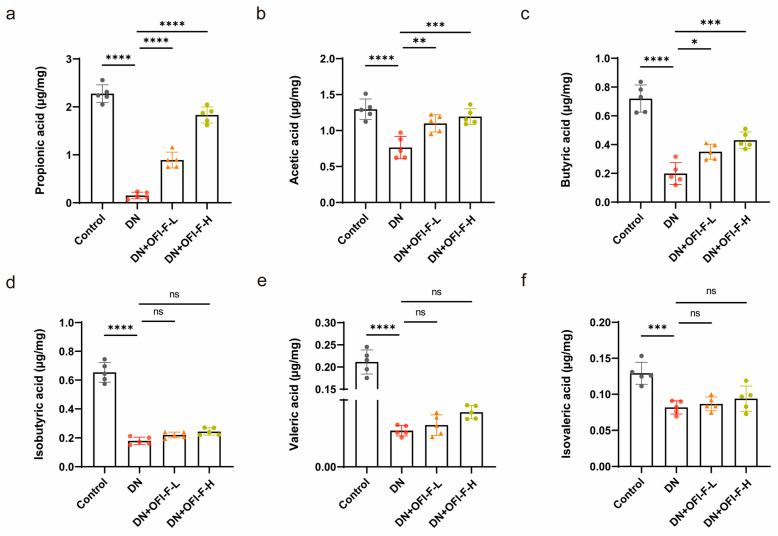
Effects of OFI-F on SCFA production in DN mice. The concentrations of propionic acid (**a**), acetic acid (**b**), butyric acid (**c**), isobutyric acid (**d**), valeric acid (**e**), and isovaleric acid (**f**) in feces of mice in four different condition groups. Data are presented as mean ± SEM (*n* = 5). Statistical significance (* *p* < 0.05, ** *p* < 0.01, *** *p* < 0.001, **** *p* < 0.0001; ns, not significant) is determined by one-way ANOVA followed by Tukey’s multiple comparison test.

In order to better understand how OFI-F influences gut microbiota, we measured the concentrations of SCFAs via GC-MS analysis. The results showed that the concentrations of propionic acid, acetic acid, butyric acid, isobutyric acid, valeric acid, and isovaleric acid in the feces of DN mice were reduced compared with the control group mice (Figure 5a–f). Interestingly, OFI-F restored the changes in propionic acid, acetic acid, and butyric acid in the feces (*p* < 0.05). However, the levels of isobutyric acid, valeric acid, and isovaleric acid did not change significantly after OFI-F treatment, but there was still an upward trend.

### 3.6. OFI-F Restored Intestinal Inflammation and Repaired Damaged Intestinal Barrier in DN Mice

**Figure 6:**
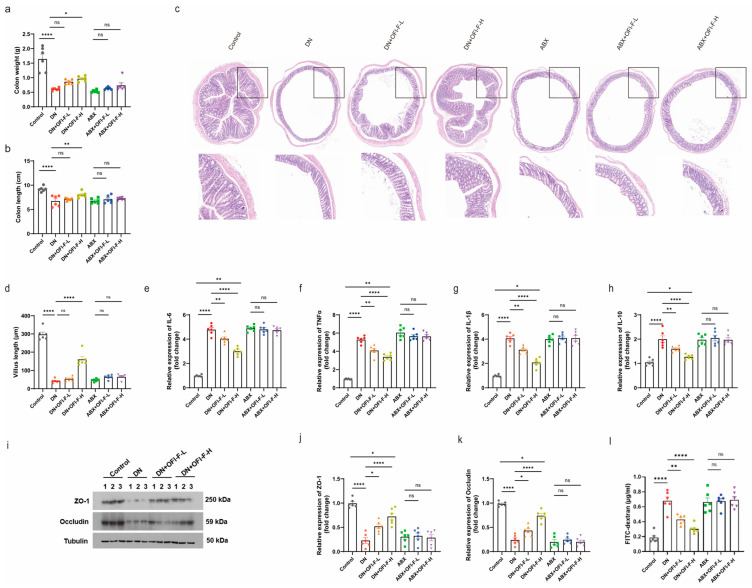
OFI-F treatment restores impaired gut barrier and improves inflammatory response in DN mice: (**a**) Average colon weight of mice. (**b**) Average colon length of mice. (**c**) Histopathological diagnosis of mouse colon using HE staining. (**d**) Villus length in the colon. The statistical results are calculated in ImageJ software. Expression levels of inflammatory factors, including *IL-6* (**e**), *IL-10* (**f**), *IL-1β* (**g**), and *TNFα* (**h**), are detected by RT-qPCR in mouse colon tissues. (**i**) Measurement of the expression levels of intestinal barrier-related proteins ZO-1 and Occludin in mouse colon tissue by immunoblotting analysis. *ZO-1* (**j**) and *Occludin* (**k**) are detected by fluorescence quantitative PCR in mouse kidney tissues. (**l**) Intestinal permeability was assessed by measuring FITC-dextran. GAPDH is selected as an internal standard. Data are presented as mean ± SEM (*n* = 6). Statistical significance (* *p* < 0.05, ** *p* < 0.01, **** *p* < 0.0001; ns, not significant) is determined by one-way ANOVA followed by Tukey’s multiple comparison test or Kruskal–Wallis test followed by Dunn’s multiple comparison test (for qPCR results).

Based on the previous observation that OFI-F can alter the gut microbiota and promote SCFA production, we hypothesized that OFI-F may further influence intestinal health and that its therapeutic effects are associated with the gut microbiota. As shown in Figure 6a,b, DN mice exhibited reduced colon weight and shorter colon lengths. HE staining showed shorter colonic villi and inflammatory cell infiltration in DN mice. All symptoms improved after OFI-F treatment compared to the model group; however, the effects of OFI-F diminished in the ABX intervention group (Figure 6c,d). These results suggest that the role of OFI-F is closely related to gut microbiota. Impaired intestinal function is closely related to intestinal barrier dysfunction and inflammation. We further found that OFI-F reduced the mRNA expression levels of pro-inflammatory factors *IL-6*, *TNFα*, *IL-1β*, and *IL-10* while increasing the expression levels of ZO-1 and Occludin in the colon of DN mice (Figure 6e–k). In addition, OFI-F treatment reduced intestinal permeability in DN mice (Figure 6l). However, the effects of OFI-F were counteracted by ABX interference. These results indicate that OFI-F treatment reduced intestinal inflammation and improved the integrity of the intestinal mucosal barrier in DN mice, suggesting that OFI-F exerts its therapeutic effect by modulating the gut microbiota.

## 4. Discussion

DN is one of the leading causes of ESRD worldwide, and it is crucial to develop new agents to control the DN process better [31,32]. OFI fruit is rich in flavonoids, but its therapeutic effect on DN is still in its preliminary stage, and the related mechanism is unknown [16]. This study investigated the effects of OFI-F on the kidney, intestinal barrier, and gut microbiota in a DN murine model. The primary objectives include the following: (1) evaluating the anti-inflammatory, antioxidant, hypoglycemic, and renal protective activities of OFI-F in vivo, and (2) investigating the effects of OFI-F on renal injury, gut microbiota, SCFAs, and the intestinal barrier in DN mice. DN is characterized by hyperglycemia and impaired renal function. Consistent with previous findings, DN model mice exhibited significantly elevated fasting blood glucose and renal index values [33,34]. Hyperglycemia is the primary clinical manifestation of diabetes mellitus and a major contributor to the development of chronic complications, including diabetic kidney disease (DKD). Excess glucose leads to an imbalance in redox states and causes both systemic and intrarenal inflammation, playing a crucial role in the pathogenesis of DKD. In obese diabetic patients, the excessive accumulation of fat induces stress in adipocytes through hyperplasia and hypertrophy. This results in hypoxia and inflammation, increases macrophage infiltration, and promotes the release of pro-inflammatory cytokines such as TNF-α, IL-6, and IL-10. These factors subsequently contribute to worsening insulin resistance [35,36]. TNF-α further exacerbates chronic hyperglycemia by stimulating the release of additional cytokines and chemokines that activate NF-κB. This signaling pathway impairs glucose uptake and perpetuates insulin resistance, sustaining a state of prolonged metabolic dysfunction [37]. Importantly, treatment with OFI-F significantly reduced blood glucose levels in DN mice, highlighting its potential as a therapeutic intervention for DN.

Recent studies have shown that DN is linked to changes in the composition or function of gut microbiota [38,39]. This study found that OFI-F may further promote its therapeutic effect on DN by promoting the production of gut microbiota and host-available SCFAs. This conception was supported by findings from ABX intervention treatments, which demonstrated that the protective effects of OFI-F on the renal and intestinal barrier were diminished following ABX treatment. Previous studies have demonstrated that the gut microbiota structure of DN patients or animal models undergo significant changes [40,41,42], which was also shown in this study. We found that OFI-F treatment was positively correlated with levels of *Bacteroides*. *Bacteroides* is one of the most important bacterial colonizers in the human gut and can stimulate SCFA production [43]. A reduction in *Bacteroides* is closely associated with inflammation [44]. Previous studies have reported that the *Bacteroidetes*/*Firmicutes* ratio (B/F ratio) was significantly reduced after renal injury [45]. Consistently, the B/F ratio of the OFI-F-treated group was significantly higher than that of the DN group. The abundance of SCFA-producing bacteria, specifically *Akkermansia*, increased in the group treated with OFI-F. *Akkermansia muciniphila* is a mucin-degrading bacterium whose increased abundance participates in a positive feedback loop, ultimately leading to the enhanced protection of host intestinal cells [46]. In addition, *Akkermansia* abundance is negatively correlated with chronic systemic inflammatory diseases [47,48,49]. OFI-F treatment reduced the abundance of *Alistipes, Desulfovibrionaceae,* and *Helicobacter* in the DN group mice. Studies have found that *Alistipes* induce inflammation and epithelial changes in hypertension [50,51]. The coexistence of early DN and *Helicobacter* infection may lead to significant proteinuria, lipid metabolism disorders, and elevated serum levels of inflammatory cytokines [52]. *Desulfovibrionaceae* has been identified in studies related to diabetes, and its abundance shows a positive correlation with the glycemic index [53].

**Figure 7:**
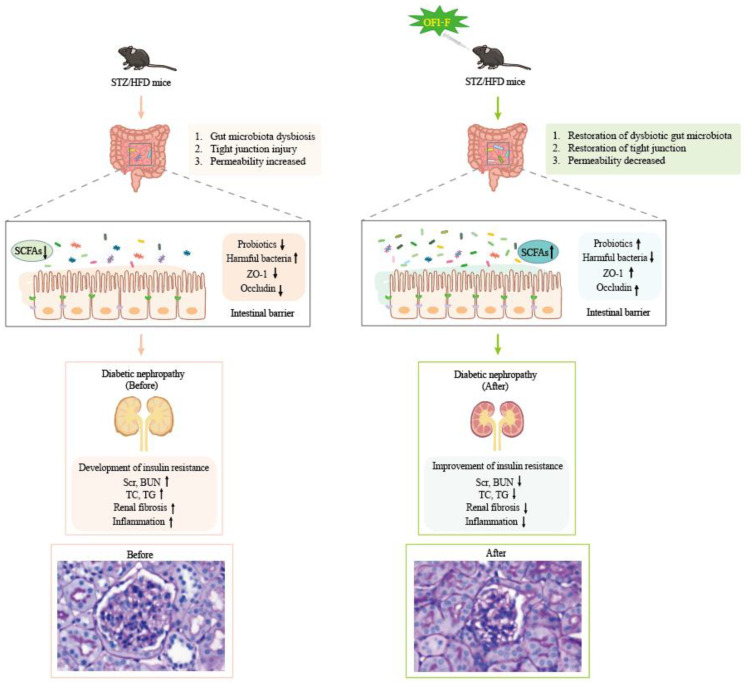
OFI-F may exert therapeutic effects on renal injury by affecting gut microbiota and its metabolites.

SCFAs are produced by microbial fermentation and are key signaling molecules in the gut–kidney axis. To explore the effect of OFI-F on SCFAs, our study showed that SCFA levels were significantly decreased in DN mice, which was consistent with previous studies on DN patients and animal models. OFI-F significantly increased fecal propionate acid, acetate acid levels, and butyrate acid, but valeric acid, isobutyrate acid, and isovaleric acid levels did not change significantly. Propionic acid, as the main fermentation product of *Bacteroides*, could prevent renal injury [54,55]. Acetic acid can reduce kidney damage [56], improve renal function [57], and reduce renal toxicity [58]. In addition, propionic acid may play a role in regulating communication between the intestine and the kidney [47]. Butyrate acid, as the main fermentation product of *Akkermansia*, can maintain tight junctions and inhibit renal injury and fibrosis, oxidative stress, and inflammatory response [59,60]. Consistent with these results, our study also demonstrated OFI-F treatment reversed intestinal barrier damage and reduced the risk of intestinal inflammation. This effect may be linked to SCFAs (see Figure 7). However, the metabolic conversion of OFI-F into various SCFAs by gut microbiota following its arrival in the intestinal lumen has not been fully examined in this study. And which signaling pathways will be affected by the significantly changed propionic acid, acetate acid, and butyric acid levels, as well as how these pathways contribute to the therapeutic effects of OFI-F, still requires further investigation.

A series of studies have shown that the sirtuin family may serve as a molecular target for the clinical exploration of new therapeutic approaches for DN. The overexpression of SIRT1, SIRT3, SIRT4, SIRT6, and SIRT7 has been found to improve abnormal manifestations in the progression of DN, such as inflammation, oxidative stress, renal fibrosis, as well as impaired cell apoptosis and autophagy, through multiple complex signaling pathways, including the SIRT1/NF-κB related pathway, TGF-β1/Smad3 pathway, and PI3K/AKT/FOXO pathway [7,10,16,61]. Moreover, previous studies have found that the flavonoid compounds isoliquiritigenin and baicalin can upregulate SIRT1 through the NF-κB and TGF-β pathways, thereby inhibiting renal inflammation and alleviating fibrosis [7]. Resveratrol is a classic activator of SIRT1. Clinical studies have demonstrated its potential therapeutic benefits for lipid profile, blood pressure, inflammation, and renal function, but it has poor pharmacokinetics and low bioavailability [62]. Based on the above reports and the findings of this study, we speculate that OFI-F may act as a potent agonist of sirtuins, ameliorating renal inflammation and fibrosis during the progression of DN by regulating the Sirtuins/NF-κB related pathway. Large-scale clinical trials and mechanistic studies are still needed to optimize the treatment strategies for DN. Moreover, there are still many limitations regarding this study, including the small experimental sample size, the use of only one DM model, the lack of pathological evaluation, the lack of in vitro experiments on the direct effects of OFI-F on the kidney, and the lack of research on various related mechanisms, which need to be further investigated in the future.

## 5. Conclusions

OFI-F improves kidney injury, alleviates intestinal inflammation, and repairs damaged intestinal barriers in DN mice by modulating the gut microbiota and increasing SCFAs levels. In vitro studies demonstrate that OFI-F treatment suppresses inflammation and oxidative stress in the LPS-induced nephritis cell model. Additionally, in vivo experiments reveal that OFI-F administration lowers blood glucose levels, improves renal function markers, attenuates oxidative stress, and alleviates renal inflammation in DN mice. OFI-F treatment increases the relative abundance of *Akkermansia* and *Bacteroidetes* and increases the levels of propionic acid, acetic acid, and butyric acid. Furthermore, through ABX experiments, we confirmed that OFI-F affects renal function and alleviates kidney injury by altering the composition and abundance of the gut microbiota. In the future, it will be necessary to increase clinical samples of DN patients and experimental animal samples, supplement the renal filtration rate and pathological damage assessment, and expand the experimental group to explore whether OFI-F has a direct effect on DN kidney damage. Additionally, further studies are needed to examine the metabolic degradation patterns of OFI-F in the digestive tract and investigate its anti-fibrotic activity both in vivo and in vitro.

## Figures and Tables

**Table 1:** Primer sequences of genes.

Gene Name	Forward	Reverse
*TNF-α*	5′-CATCTTCTCAAAATTCGAGTGACAA-3′	5′-TGGGAGTAGACAAGGTACAACCC-3′
*IL-6*	5′- AACGATGATGCACTTGCAGA -3′	5′- GAGCATTGGAAATTGGGGTA -3′
*IL-1β*	5’-GATCCACACTCTCCAGCTGCA-3’	5’-CAACCAACAAGTGATATTCTCCATG-3’
*GADPH*	5′-TGGCAAAGTGGAGATTGTTGC-3′	5′-AAGATGGTGATGGGCTTCCCG-3′
*IL-10*	5’-ACCTGCTCCACTGCCTTGCT-3’	5’-GGTTGCCAAGCCTTATCGGA-3’
*TLR4*	5′-ATGGCATGGCTTACACCACC-3′	5′- GAGGCCAATTTTGTCTCCACA-3′
*MYD88*	5′-TCATGTTCTCCATACCCTTGGT-3′	5′-AAACTGCGAGTGGGGTCAG-3′
*NF-κB*	5′-ATGGCAGACGATGATCCCTAC-3′	5′-TGTTGACAGTGGTATTTCTGGTG-3′
*COL1A1*	5′-GAGAACCAGCAGAGCCA-3′	5′-GAACAAGGTGACAGAGGCATA-3′
*YAP*	5′-CTGGGTGCCGTTTCTCCTG-3′	5′-CCATGTTGTTGTCTGATCGTTG-3′
*TGF-β*	5′-CCGCAACAACGCCATCTA-3′	5′-ACCAAGGTAACGCCAGGAAT-3′
*ZO-1*	5′-AGCTCATAGTTCAACACAGCCTCCAG-3′	5′-TTCTTCCACAGCTGAAGGACTCACAG-3′
*Occludin*	5′-GGACCCTGACCACTATGAAACAGACTAC-3′	5′-ATAGGTGGATATTCCCTGACCCAGTC-3′

**Table 2:** Primer sequences of bacteria.

Bacteria Name	Forward	Reverse
Bacteroidota	5′-TCCTACGGGAGGCAGCAGT-3′	5′-CAATCGGAGTTCTTCGTG-3′
Desulfobacterota	5′-GTCCACGCCGCCTATGGGGTGCAGCAGTG-3′	5′-GTCCATTGCCGGACTACAAGGGTATCTAATCCTGTTTG-3′
Akkermansia	5′-CAGCACGTGAAGGTGGGGAC-3′	5′-CCTTGCGGTTGGCTTCAGAT-3′
Helicobacter	5′-GCCTGGGGAGTTTATGGAGAGTTTGATCCTGGC-3′	5′-ATCCTCCGTAAAGGAGGTGATCCAGCCG-3′
Alistipes	5′-GCGTGGGGAGTACGGAGGATTCAAGCGTTATCC-3′	5′-ATCCTCCGTACTCCCCACGCTTTCGTGC-3′

## Data Availability

The original contributions presented in this study are included in this article, and further inquiries can be directed to the corresponding author.

## Supplementary Materials

The following supporting information can be downloaded at: https://www.mdpi.com/article/10.3390/nu17111800/s1, Figure S1: OFI-F extraction flow chart; Figure S2: CCK8 experiments with different concentrations of OFI-F treatment in Hkb-20 cells; Figure S3: (a) Average daily water intake per mouse (*n* = 6). (b) Average daily dietary intake per mouse (*n* = 6).
