# Supplementary material for: Flavonoids Derived from Opuntia ficus-indica Fruit Alleviate Renal Injury in Diabetic Nephropathy Mice by Altering Gut Microbiota and Promoting the Production of SCFAs
Source: Nutrients. 2025 May 26;17(11):1800. doi: 10.3390/nu17111800 (PMC12157904; doi:10.3390/nu17111800)
Supplement: Supplementary file 1 [file nutrients-17-01800-s001.zip › nutrients-3624191-supplementary.pdf]

Flavonoids derived from *Opuntia ficus-indica* fruit alleviates renal injury in diabetic nephropathy mice by altering gut microbiota and promoting the production of SCFAs

Haiping Liao[1†], Yunyi Zhao[1†*], Yongheng Liang[1], Kang Zou[2]

[1] College of Life Sciences, Key Laboratory of Agricultural Environmental Microbiology of Ministry of Agriculture and Rural Affairs, Nanjing Agricultural University, Nanjing, China.

[2] College of Animal Science and Technology, Nanjing Agricultural University, Nanjing, 210095, China; Stem Cell Research and Translation Center, Nanjing Agricultural University, Nanjing, 210095, China.

*Corresponding author(s). E-mail(s): yunyizhaodd@163.com

Contributing authors: liaohaiping216@163.com; liangyh@njau.edu.cn

† These authors contributed equally to this work.

Electronic supplementary material

Supplementary data

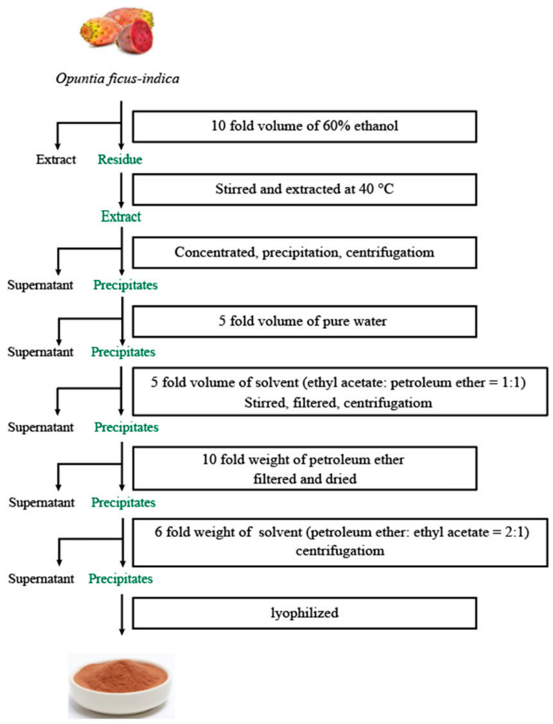

Figure S1. OFI-F extraction flow chart.

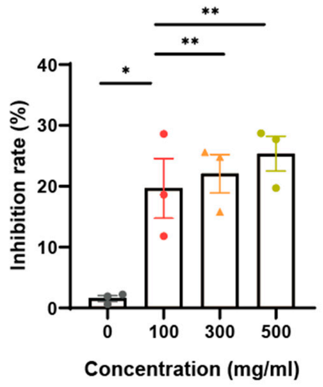

Figure S2. CCK8 experiments with different concentrations of OFI-F treatment in Hkb-20 cells. According to the CCK8 cell inhibition criterion, in the range of 1-20%, the drug treatment did not inhibit or slightly inhibited cell proliferation.

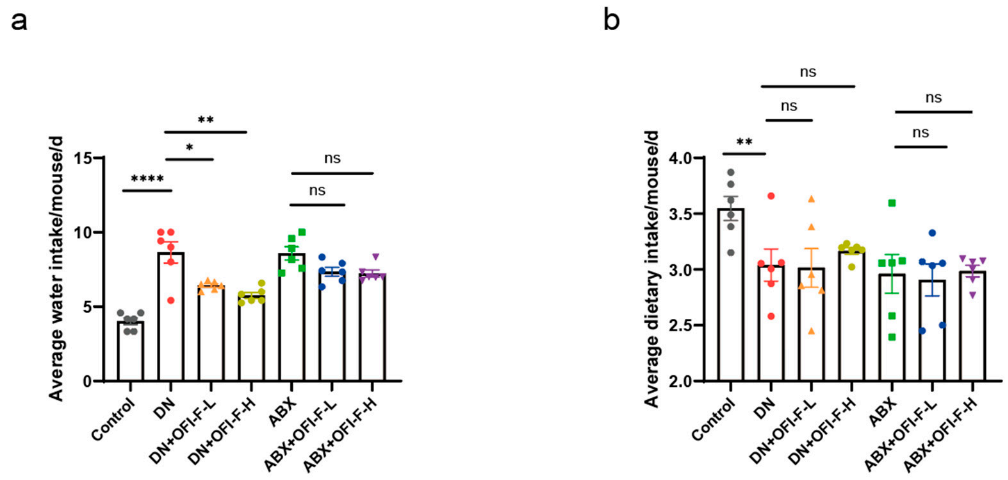

Figure S3. (a) Average daily water intake per mouse (n=6). (b) Average daily dietary intake per mouse (n=6).
